# Characterization of disease-specific cellular abundance profiles of chronic inflammatory skin conditions from deconvolution of biopsy samples

**DOI:** 10.1186/s12920-019-0567-7

**Published:** 2019-08-17

**Authors:** Zandra C. Félix Garza, Michael Lenz, Joerg Liebmann, Gökhan Ertaylan, Matthias Born, Ilja C. W. Arts, Peter A. J. Hilbers, Natal A. W. van Riel

**Affiliations:** 1 Department of Biomedical Engineering, Eindhoven University of Technology, Eindhoven, The Netherlands; 2 Department of Medical Microbiology, Academic Medical Center, University of Amsterdam, Amsterdam, The Netherlands; 3 Maastricht Centre for Systems Biology (MaCSBio), Maastricht University, Maastricht, The Netherlands; 4 Faculty of Biology, Institute of Organismic and Molecular Evolution, Johannes Gutenberg University Mainz, Mainz, Germany; 5 Preventive Cardiology and Preventive Medicine – Center for Cardiology, University Medical Center of the Johannes Gutenberg University Mainz, Mainz, Germany; 6 Philips Electronics Netherlands B.V., Research, Eindhoven, The Netherlands; 7 VITO Health, VITO NV, Mol, Belgium

**Keywords:** Skin, Epidermis, Dermis, Chronic inflammatory skin diseases, Leukocytes, Microarrays, Gene expression

## Abstract

**Background:**

Psoriasis and atopic dermatitis are two inflammatory skin diseases with a high prevalence and a significant burden on the patients. Underlying molecular mechanisms include chronic inflammation and abnormal proliferation. However, the cell types contributing to these molecular mechanisms are much less understood. Recently, deconvolution methodologies have allowed the digital quantification of cell types in bulk tissue based on mRNA expression data from biopsies. Using these methods to study the cellular composition of the skin enables the rapid enumeration of multiple cell types, providing insight into the numerical changes of cell types associated with chronic inflammatory skin conditions. Here, we use deconvolution to enumerate the cellular composition of the skin and estimate changes related to onset, progress, and treatment of these skin diseases.

**Methods:**

A novel signature matrix, i.e. DerM22, containing expression data from 22 reference cell types, is used, in combination with the CIBERSORT algorithm, to identify and quantify the cellular subsets within whole skin biopsy samples. We apply the approach to public microarray mRNA expression data from the skin layers and 648 samples from healthy subjects and patients with psoriasis or atopic dermatitis. The methodology is validated by comparison to experimental results from flow cytometry and immunohistochemistry studies, and the deconvolution of independent data from isolated cell types.

**Results:**

We derived the relative abundance of cell types from healthy, lesional, and non-lesional skin and observed a marked increase in the abundance of keratinocytes and leukocytes in the lesions of both inflammatory dermatological conditions. The relative fraction of these cells varied from healthy to diseased skin and from non-lesional to lesional skin. We show that changes in the relative abundance of skin-related cell types can be used to distinguish between mild and severe cases of psoriasis and atopic dermatitis, and trace the effect of treatment.

**Conclusions:**

Our analysis demonstrates the value of this new resource in interpreting skin-derived transcriptomics data by enabling the direct quantification of cell types in a skin sample and the characterization of pathological changes in tissue composition.

**Electronic supplementary material:**

The online version of this article (10.1186/s12920-019-0567-7) contains supplementary material, which is available to authorized users.

## Background

Chronic inflammatory skin diseases like psoriasis (Ps) and atopic dermatitis (AD) have a high prevalence [1] and often pose a burden to the patients [2, 3]. The journey of patients with psoriasis and atopic dermatitis to get the right treatment is lengthy [4]. Lesions of psoriasis and atopic dermatitis are characterized by the increased proliferation and abnormal differentiation of keratinocytes [5], and the presence of immune infiltrates like T cells and dendritic cells (DCs) [6, 7]. However, different subsets of immune infiltrates are associated with Ps and AD [7, 8].

Psoriasis is driven by the IL-23/ Th17 T cell axis [7], in which Th17 and Tc17 induce the release of cytokines that lead to an inflammatory response that self-amplifies and results in the recruitment of Th1 and Th22 T cells. AD in contrast, is driven by Th2 and Th22 [7]. Cytokines secreted by these cell types induce a decrease in the terminal differentiation of keratinocytes and contribute to the distinctive epidermal barrier dysfunction [7]. Psoriasis and atopic dermatitis have distinct clinical manifestations. Psoriasis is characterized by well-defined areas of thick, dry, flaky, red skin [6]; typically on the elbows, knees, and scalp [3]. Features of lesions in atopic dermatitis include patches of highly pruritic, eczematous erythematous skin with scratch marks and serous exudation; commonly observed on the face, flexures, and wrists [9]. To better understand the underlying mechanisms of these two chronic inflammatory skin diseases, studies have explored the mRNA expression profiles of lesional, non-lesional [10–12] and healthy skin [13], leading to the identification of differentially expressed genes. However, the interpretation of mRNA expression data from the skin remains a critical challenge due to the large heterogeneity in the samples, much affected by the site and method of sample acquisition, as well as the lack of good markers for several of the specialized cell types found in this tissue. Previous investigations have sought to identify the gene expression signature of skin-specific cell types, structures, and processes [14, 15]. Swindell et al. [14] observed a range of inflammatory and cytokine-related gene expression patterns in psoriasis patients. They identified 1233 differentially expressed genes increased in psoriasis lesions, which were attributed to keratinocyte activity, infiltration of T cells and macrophages. They also observed an increased inflammatory signature in 50% of the patients, with increased expression of genes expressed by T cells, monocytes, and dendritic cells. Shih et al. [15] derived expression signatures for adipocytes, fibroblasts, melanocytes, macrophages/dendritic cells, T cells, keratinocytes, appendages and core processes of the skin. They applied their set of skin-specific expression signatures to the analysis of transcriptomic datasets from 18 dermatological conditions, including psoriasis and atopic dermatitis and showed the over- and under-representation of various cell types in skin diseases based on the reduction or upsurge of their defined gene signatures. The resources provided by these investigations aid in the interpretation of expression data derived from human skin and enable the study of tissue composition. Nevertheless, they do not directly quantify the cell-types and tissue-specific variations in a given skin sample. Further work is therefore needed to enumerate the abundance of skin-related cell types in common chronic inflammatory skin conditions.

Currently, changes in cellular composition of a given tissue are experimentally observed by flow cytometry [16, 17] and immunohistochemistry [18, 19] studies. However, these methods are constrained by the availability of cell type-specific biomarkers and are restricted to identification of a small subset of cell types. Computational methodologies, like deconvolution, have been developed for predicting the relative fractions of multiple cell types in a sample solely based on gene expression profiles [20, 21]. These approaches enable the large-scale analysis of mRNA mixtures for identifying cellular biomarkers and novel therapeutic targets without the need for experimental techniques [22]. Deconvolution methods, like CIBERSORT [23], infer the abundance of various cell types in a sample using a reference dataset and the mRNA expression profile from the sample of interest [24]. Deconvolution techniques have been successfully used to determine the composition of immune cell types in bulk tissue [25], but have not been applied yet to chronic inflammatory skin disorders. Using deconvolution methods to study the cellular composition of the skin would allow the simultaneous quantification of multiple cell types, providing additional insight into the relative abundance changes of specific cell types that occur at the onset, development, and treatment stages of dermatological diseases.

Here, we performed the computational deconvolution of layer-specific and whole skin transcriptomics data into cell type-specific fractions. We analyzed mRNA expression profiles from samples of isolated epidermis and dermis as well as whole skin biopsies of 114 healthy subjects, 241 psoriasis patients, and 38 patients with atopic dermatitis. We quantified the cellular composition of the skin and estimated the changes associated with the onset, progress, and treatment of Ps and AD. This resource aids in the interpretation of transcriptomics data derived from skin by allowing the direct enumeration of cell types in a skin sample and enabling the characterization of pathological changes in tissue composition associated with chronic inflammatory skin conditions.

## Methods

### Aim, design, and setting

In this study, the CIBERSORT [23] deconvolution algorithm is used together with a novel signature matrix, termed DerM22, containing all major cell types that are present in the skin. The approach is applied to publicly available microarray mRNA expression data from healthy skin as well as lesional and non-lesional skin samples from patients with psoriasis or atopic dermatitis. The methodology is validated by comparison to experimental results from flow cytometry and immunohistochemistry reported in the literature as well as by application to independent data from isolated cell types.

### Signature matrix definition and cell frequency estimation

The CIBERSORT deconvolution algorithm uses nu-support vector regression together with a signature matrix of cell type-specific mRNA expression to estimate the cell type composition of tissue samples. The focus of the original work is on immune cell types to estimate the cellular composition of blood as well as the immune cell content in certain cancers. Recently, we have generated a novel signature matrix, called AT21, for deconvolution of the adipose tissue cell types [26]. Here, we have extended the AT21 signature matrix using two publicly available datasets containing keratinocyte mRNA expression (GEO identifiers GSE30355 [27] and GSE36287 [28]), ensuring its applicability to the deconvolution of skin samples. The resulting DerM22 signature matrix (Additional file 1: Table S2 and Additional file 2: Figure S1) contains cell type-specific signatures of 22 isolated cell types and is based on a reference dataset of 220 samples from 22 original studies Additional file 3: Table S3).

For the generation of DerM22, raw data (CEL-files) of the 22 original studies (all hybridized to the Affymetrix HG-U133 Plus 2.0 microarray platform) were downloaded from Gene expression omnibus (GEO) database and preprocessed with Affymetrix Power Tools (https://www.thermofisher.com/nl/en/home/life-science/microarray-analysis/microarray-analysis-partners-programs/affymetrix-developers-network/affymetrix-power-tools.html#) using the robust multi-array average (RMA) normalization method. Then, the dataset was uploaded to the CIBERSORT website (https://cibersort.stanford.edu) for probe filtering based on the following three steps: (i) select probes that are differentially expressed between any individual cell type and all other samples (q value < 0.3 (false discovery rate), two-sided unequal variance t-test); (ii) rank probes according to their fold change between the respective cell type and all other samples; and (iii) include the top G probes per cell type in the signature matrix, where G (between 50 and 150) is selected to minimize the condition number of the signature matrix [23]. This resulted in a selection of 2045 probes included in DerM22, which are represented by the average probe intensity value for each of the 22 cell types.

### Datasets

The deconvolution approach is applied to various public datasets from two different Affymetrix microarray platforms. The main analysis is performed on datasets from the Affymetrix HG-U133 Plus 2.0 platform (GPL570 in GEO), which was chosen to avoid any cross-platform issues with the reference dataset used to generate DerM22. This data includes a dataset with measured expression of different skin layers that were separated by laser capture microdissection [29] (GSE42114) and 1006 whole-skin 4–6 mm punch biopsy samples from healthy people and individuals with psoriasis or atopic dermatitis (Table 1).

**Table 1:** Skin biopsy-derived microarray datasets used for deconvolution analysis

Skin disease	Accession number	Samples	Location of sample acquisition	Reference
Psoriasis	GSE13355	180	Lesional samples: trunk or upper/lower limbs. Healthy and non-lesional samples: buttock or upper thigh area	[30]
	GSE30999	170	Lesional samples: trunk or upper/lower limbs. Healthy and non-lesional samples: anatomical region similar to that of the lesional sample.	[31]
	GSE34248	28	Lesional samples: unspecified. Healthy and non-lesional samples: anatomical region similar to that of the lesional sample.	[32]
	GSE41662	48		[32]
	GSE78097	33	Lesional samples: trunk, upper/lower limbs, scalp, and palmoplantar areas. Healthy and non-lesional samples: anatomical region similar to that of the lesional sample.	[33]
	GSE14905	82	Lesional samples: trunk or upper/lower limbs. Healthy and non-lesional samples: anatomical region similar to that of the lesional sample.	[34]
	GSE47751	34	Lesional and non-lesional samples: dependent on the location of the lesion.	[35]
	GSE117239	324	Lesional and non-lesional samples: trunk and extremities.	[36]
Atopic dermatitis	GSE27887	35	Lesional samples: dependent on the location of the lesion. Healthy and non-lesional samples: anatomical region similar to that of the lesional sample.	[37]
	GSE32924	33		[10]
	GSE36842	39		[38]

Datasets from a second microarray platform (Affymetrix HG-U133A, GPL96 in GEO) is used to test the cross-platform performance of the algorithm as well as for validation of the deconvolution approach using isolated cell types. The datasets contain skin samples from psoriasis (study GSE6710) [39], as well as several isolated cell types that were used to build the original LM22 signature matrix as published in [23] (studies E-MEXP-750, GSE22886, GSE4527, GSE5099, GSE7138, and data from [40, 41]). Furthermore, samples from isolated keratinocytes (studies GSE26688 [42] and GSE6932 [43]), epidermal stem cells, and transit amplifying cells (study GSE4858) [44] are also included.

For both microarray platforms, raw data (CEL-files) were downloaded and RMA-normalized with Affymetrix Power Tools as described above.

### Validation of the approach

We used four different ways of assessing the performance of DerM22 in deconvoluting skin samples, namely (i) comparison of cell fractions to experimental results from flow cytometry analysis reported in the literature, (ii) comparison of ratios of two cell counts between two phenotypic conditions to literature reports from immunohistochemistry analysis, (iii) cross-platform analysis of isolated cell types from independent datasets, and (iv) comparison of deconvolution of datasets from multiple original studies to test the reproducibility of results.

### Flow cytometry data

A manual (non-systematic) literature review was performed to identify quantitative reports about the cellular composition of the human skin. A total of 5 original studies that report cell type-specific cell counts in human skin were selected. Information about the cell type, disease state, number of individuals, and the marker used for cell counting was extracted along with the mean and standard error (where available) of reported counts (Additional file 4: Table S1).

### Immunohistochemistry ratios

**Fig. 1:**
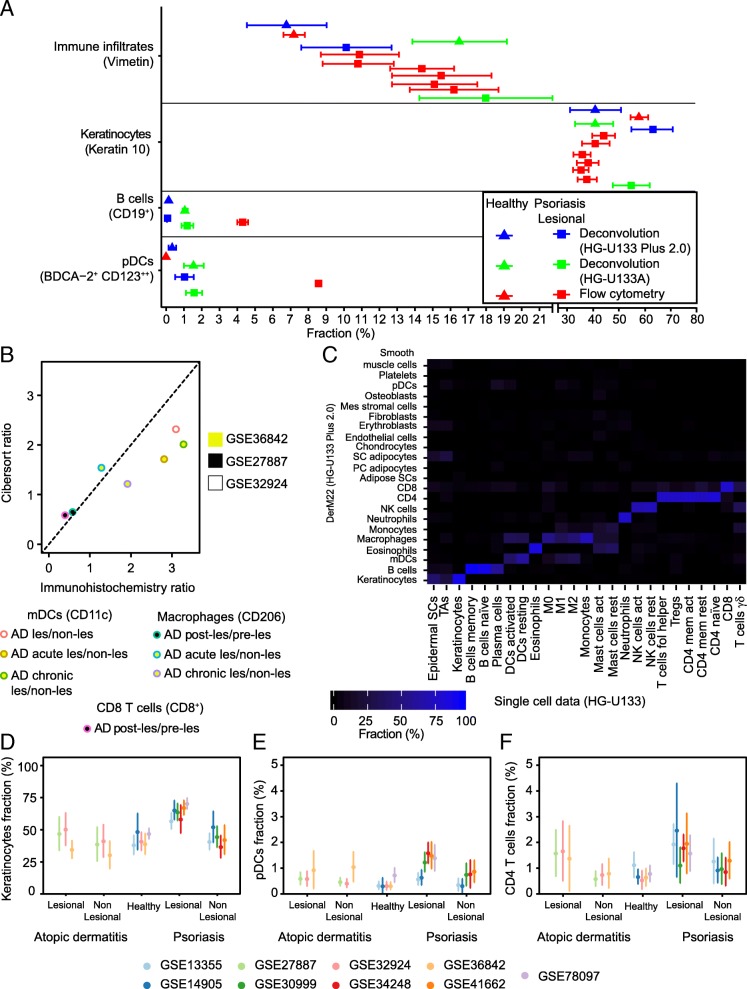
Performance assessment of signature matrix DerM22 for deconvolution of gene expression data from skin biopsies. **a** The accuracy of DerM22 is analyzed by comparing the mean deconvolution results (blue) to mean flow cytometry data (red) for samples of healthy subjects (triangle), and the lesional skin of psoriasis patients (square). For the flow cytometry data and CIBERSORT results, the standard error is shown for all cell subsets except for the flow cytometry-derived psoriasis pDCs values given that it was not reported in the original study. Also, the cross-platform bias is explored by the comparison of the deconvolution results (blue) from microarray data acquired in the same platform as DerM22, i.e. Affymetrix HG-U133 Plus 2.0, to data obtained using a different platform (green), i.e. Affymetrix HG-U133A. **b** The performance of DerM22 is also compared to immunohistochemistry results from three different studies, i.e. GSE36842 (yellow), GSE27887 (black), GSE32924 (white), for which expression data is also available. This is done by deriving the ratio of average myeloid dendritic cells (CD11c), macrophages (CD206), and CD8^+^ T cells in lesional to non-lesional skin, or in treated to untreated lesional skin of atopic dermatitis (AD). The plotted ratios correspond to the ratios of the average for each subgroup. **c** The cross-platform bias is further analyzed by deconvolving expression data from 25 different isolated cell types acquired with Affymetrix HG-U133A. The estimated fractions of each cell type are presented in a heatmap, ranging from 0% (black) to 100% (bright blue). **d**-**f** The consistency of the mean results (+/− standard deviation) across independent datasets of skin in health and disease is examined for CD4^+^ T cells, plasmacytoid dendritic cells (pDCs), and keratinocytes. For results on other cell types see Additional file 5: Fig. S2

Three of the studies included in our deconvolution analysis [10, 37, 38] also reported immunohistochemistry-based cell counts of myeloid dendritic cells (mDCs), macrophages, or CD8^+^ T Cells (Fig. 1b). These counts are, however, usually reported as cell count per area of skin tissue and are difficult to convert into fractional cell counts, i.e. number of cells of the respective cell type per total number of cells. Therefore, we calculated ratios of average cell counts between lesional and non-lesional skin samples or between skin samples from before and after UV-treatment. We used these ratios for comparison of our CIBERSORT-based estimates to the immunohistochemistry values.

### Cross-platform comparison

The independent cross-platform validation of the skin deconvolution using DerM22 was performed based on the Affymetrix HG-U133A dataset. Probe matching was performed via the biomaRt R package. Subsequently, the percentages of all 22 cell types from the DerM22 signature matrix were calculated and compared to the known isolated cell type of the respective samples (Fig. 1a and c).

### Inter-study variability

For an evaluation of the reproducibility of skin cell composition estimates, the means and standard deviations of the nine original studies were compared per cell type (Fig. 1d and Additional file 5: Figure S2).

### Statistics

Non-parametric (Wilcoxon) tests were used to account for the skewed distributions of relative cell type composition and the limited range of possible values between 0 and 1. For comparisons with repeated measures within the same person (i.e. lesional vs. non-lesional or before vs. after treatment), the Wilcoxon signed rank test was used. Non-paired comparisons (diseased versus healthy, mild versus severe psoriasis) were performed using the Wilcoxon rank sum test. Tests are considered significant for *p*-values smaller than 0.05 (unadjusted or Benjamini-Hochberg adjusted, as indicated in the text). All reported p-values are without correction for multiple testing, if not stated otherwise.

## Results

### Verification of skin-specific signature matrix

To assess the performance of DerM22, we compared our deconvolution results with experimental observations reported in the literature from flow cytometry studies (Additional file 3: Table S3) of healthy skin and lesional skin of psoriasis (Fig. 1a). For this comparison, we derived the relative fraction of keratinocytes and immune infiltrates from eight independent datasets (studies GSE13355, GSE14905, GSE30999, GSE34248, GSE41662, GSE78097, GSE32924, GSE36842, described in Table 1) [10, 30–34, 38]. The average relative abundance derived from the deconvolution analysis was close to the average results of the flow cytometry studies focused on healthy and lesional psoriasis total immune infiltrates [45–47], and healthy plasmacytoid dendritic cells (pDCs) [48]. The predicted relative abundance of keratinocytes was underestimated for healthy skin and overestimated for lesional psoriasis skin compared to Keratin 10 [45–47]. However, Keratin 10 is a marker of differentiated keratinocytes only and does not account for those with the ability to proliferate [43], i.e. epidermal stem cells and transit amplifying cells [47, 49]. Therefore, this discrepancy suggested that the keratinocytes category in our approach accounts for both the differentiated and proliferating keratinocytes. Further, our method predicted a lower amount of B cells and plasmacytoid dendritic cells in lesional psoriasis than the one observed in the flow cytometry studies [48, 50]. Next, we assessed the cross-platform performance of the algorithm by performing the deconvolution of lesional psoriasis expression data acquired with Affymetrix HG-U133A (study GSE6710) [39]. In comparison to the same platform-results, the predicted relative fraction of the cross-platform immune infiltrates, B cells, and pDCs was overestimated in health and disease. The average of the predicted fractions for cross-platform keratinocytes were close to the average values from the same platform-results.

Limited by the absence of literature, the verification with flow cytometry data was not possible for atopic dermatitis. However, the three studies included in our analysis [10, 37, 38] also described immunohistochemistry-based cell counts of mDCs, macrophages, and CD8^+^ T cells (Fig. 1b). To verify our predicted estimates for atopic dermatitis, we compared our deconvolution-based relative fractions to the average immunohistochemistry values based on the ratio between lesional and non-lesional skin samples or between before and after phototherapy (Fig. 1b). All average fractions per groups from our deconvolution estimates were close in value to the ratios obtained from the immunohistochemistry cell counts. The immunohistochemistry-based ratio of myeloid dendritic cells was approximate to 3 for all three data points, while the deconvolution-based ratio was around 2. For the macrophages, both methodologies suggested a greater spread in the ratio of lesional to non-lesional and phototherapy treated lesional skin. The deconvolution-based ratios calculated for macrophages and CD8^+^ T cells were in close agreement with the immunohistochemistry-based values. These results suggested that the amount of CD8^+^ T cells and macrophages decreases with treatment. Further, our observations indicated that the amount of macrophages and dendritic cells is higher in the lesional skin of patients with atopic dermatitis compared to their non-lesional skin, regardless of the disease state.

Based on the observations from the cross-platform deconvolution results, we questioned whether expression data of isolated cell types from a different platform (i.e. Affymetrix platform HG-U133A) would be adequately assigned to the right cellular subset using DerM22. Thus, we deconvolved data from the isolated 22 leukocyte subsets included in the signature matrix LM22 developed by Newman et al. [23] and expression data from keratinocytes [43], epidermal stem cells and transit amplifying cells [44] (Fig. 1c). LM22 comprised data on 11 major leukocyte types, i.e. B cells, dendritic cells, eosinophils, monocytes and macrophages, mast cells, polymorphonuclear cells, natural killer cells (NK), plasma cells, CD4^+^ T cells, CD8^+^ T cells, and γδ T cells. As expected, with the exception of CD8^+^ cells, data of isolated cell types included in DerM22 was accurately categorized, including subsets and activation states of these cell types, despite the cross-platform differences. The CD8^+^ cells in DerM22 included mainly CD8^+^ cells, but also other T cell subsets, like γδ T cells and T helpers, and other immune cells. Nevertheless, the overlap of CD8^+^ cells with the latter was close to zero. Further, the data on isolated epidermal subsets, i.e. epidermal stem cells, transit amplifying cells, and keratinocytes were all categorized as keratinocytes. This result confirmed that the keratinocytes category in our method accounts for both proliferating and differentiated keratinocytes. The expression data from the epidermal stem cells and transit amplifying cells was also partially classified as adipocytes, suggesting that these epidermal subsets may share some similarities regarding expression signatures with the adipocytes. Despite the good performance of DerM22 when used to deconvolve expression data from a different platform, one should be cautious as cross-platform bias remains a potential error factor.

It is known that expression data is prone to significant variations depending on the site or method of acquisition [15]. Thus, we assessed the consistency of the mean deconvolution results for each cell type comprised in DerM22 across nine independent public datasets. The panels D to F of Fig. 1 depict the relative fraction of keratinocytes, plasmacytoid dendritic cells, and CD4^+^ T cells inferred from the samples of healthy skin and non-lesional and lesional skin of patients with psoriasis and atopic dermatitis in each independent study. The results for the rest of the cellular subsets in DerM22 are presented in the Additional file 5: Figure S2. The standard deviation was calculated for each mean relative fraction. The estimated abundance of cellular subsets of atopic dermatitis and healthy skin were close in value for all datasets. In the psoriasis datasets, a wider spread was observed despite the similarity in the protocol and target group.

### Deconvolution of layer-specific expression from healthy skin

**Fig. 2:**
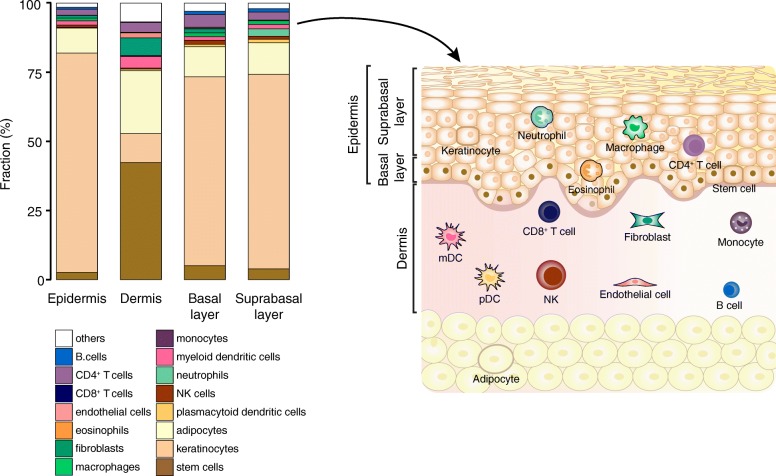
Deconvolution of the layers and sublayers of healthy skin. The results of the deconvolution of layer-specific healthy skin samples are described by the stacked bars and spatially represented in the schematic of the skin. DerM22 was used to obtain these results, which were then combined into 16 main types before analysis

After our verification, we used the signature matrix DerM22 to analyze mRNA expression data extracted from the epidermal and dermal skin layers, as well as the basal and suprabasal epidermal sublayers, from healthy subjects (Fig. 2). The deconvolution of layer-specific expression data estimated the relative abundance of keratinocytes, fibroblasts, endothelial cells, adipose stem cells, adipocytes, and leukocytes in the skin layers. Our results suggested that keratinocytes account for 79% of the cells in the epidermis. The fraction of immune cells in the epidermis was predicted to be close to 9%. The main immune cells estimated for the epidermis were CD4^+^ T cells, macrophages, neutrophils, dendritic cells, and natural killer cells. The percentage of CD8^+^ T cells was estimated to be lower than 1%. Unexpectedly, the missing 12% of the cell types estimated for the epidermis corresponds to adipocytes (10%) and adipose stem cells (2%), which were observed in both the basal and suprabasal epidermal sublayers. The presence of adipocytes and adipose stem cells was also predicted for the dermis (23 and 42%, respectively). Other cell types allocated to the dermis were the fibroblasts (7%), endothelial cells (2%), myeloid dendritic cells (4%), and CD4^+^ T cells (4%). These layer-specific profiles of healthy skin provide a general overview of the proportion in which each cell type is expected to be observed in the epidermis and dermis.

### Estimation of cell type relative abundance profiles for healthy skin and lesional and non-lesional skin of psoriasis and atopic dermatitis

**Fig. 3:**
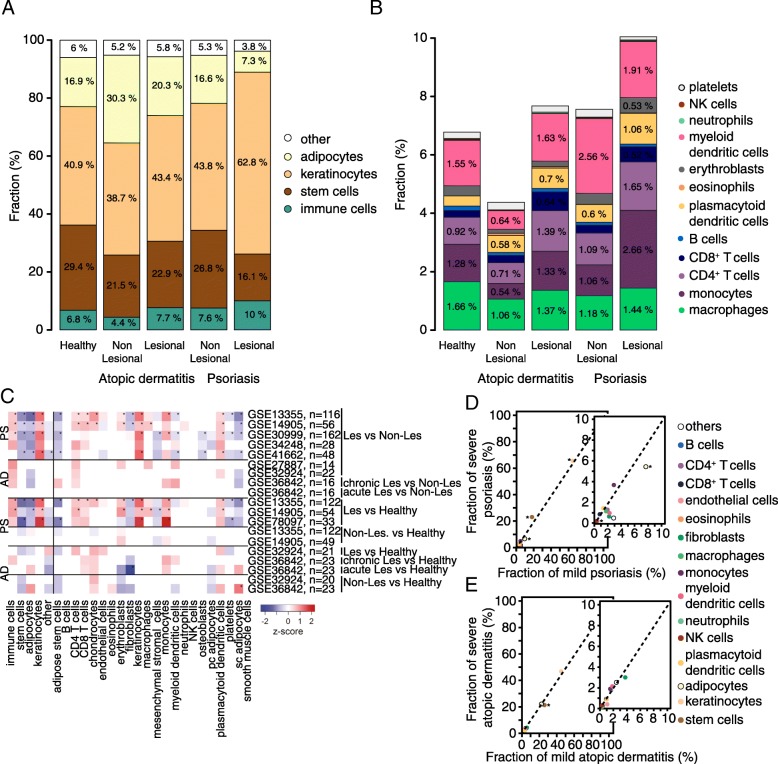
Deconvolution of skin biopsies from healthy subjects and patients with psoriasis and atopic dermatitis. **a** Comparison of the average cellular abundance derived from gene expression data of 648 biopsy samples from the skin of healthy subjects and patients with atopic dermatitis and psoriasis. The samples from psoriasis and atopic dermatitis account for lesional and non-lesional skin. The average cellular abundance is combined into five groups, i.e. immune cells (turquoise), stem cells (brown), keratinocytes (light brown), adipocytes (light yellow), and others (white). **b** Detailed description of average values in the fractions of immune infiltrates across healthy and disease skin phenotypes (depicted in the donut chart from panel **a**). **c** Heatmap of significant over (red) or under (blue) represented cell types comparing lesional to non-lesional and healthy skin samples. Non-significant results (uncorrected *p* > 0.05) are kept blank. Significant results after correction for multiple testing are labeled with a star. **d**-**e** Comparison of the estimated cell-type specific fractions for mild (x-axis) and severe (y-axis) cases of psoriasis and atopic dermatitis. The diagonal in each plot indicates the point at which the estimated fraction for a given cell type for the mild case would be equal to the one of the severe cases. The deviation from the diagonal indicates the difference between mild and severe cases of psoriasis and atopic dermatitis. Cell types with a corrected *p*-value lower than 0.05 are indicated with an asterisk

Next, we investigated whether the relative fractions derived from the deconvolution of whole skin- expression data would reflect the structural changes and the inflammatory hallmark that characterizes the lesions of psoriasis and atopic dermatitis (Fig. 3a-c and Additional file 6: Figure S3). Results of healthy skin biopsies (Fig. 3a) suggested that 40.9% of the cells in the samples correspond to keratinocytes, while 29.4% was allocated to adipose stem cells, 16.9% to adipocytes, 6.8% to leukocytes, and 6% to other cell types. Note that in contrast with the expression data used to compute the layer-specific composition of the skin described in Fig. 2, the data from the skin samples considered in this section comprises information from all the skin layers at once. Compared to healthy skin, our results showed a marked and significant increase in the abundance of keratinocytes (+ 21.9 percentage points (pp)) and immune cells (+ 3.2 pp), and a decrease in adipose stem cells (− 13.3 pp) and adipocytes (− 9.6 pp) in the psoriasis lesions (Fig. 3a, c). The immune cells that dominated this increase are monocytes (+ 1.41 pp), CD4^+^ and CD8^+^ T cells (+ 0.73 pp. and + 0.24 pp., respectively), and dendritic cells (+ 1.06 pp) (Fig. 3b-c). In the lesional skin of atopic dermatitis, we observed on average a 2.5 percentage points increase in the fraction of keratinocytes and 3.4 pp. in the relative abundance of adipocytes compared to healthy skin (Fig. 3a). We could, however, not establish statistical significance for these comparisons (Fig. 3c). Similar to the lesions of psoriasis, the lesional skin of atopic dermatitis showed on average an increase in the percentage of immune cells (+ 0.9 pp) and a decrease in the relative abundance of adipose stem cells (− 6.5 pp) compared to healthy skin (not significant). However, the predicted abundance of immune cells was on average higher in the lesional skin of psoriasis compared to atopic dermatitis. Adipocytes were predicted to be lower in lesional skin compared to non-lesional skin, with the lowest fractions estimated for lesions of psoriasis.

In addition to the general profiling of the changes in cellular subsets related to the lesional and non-lesional skin of psoriasis and atopic dermatitis, we explored the changes in cell type composition that may distinguish severe from mild cases of these dermatological disorders. For this analysis, we considered 13 samples for severe and 14 for mild psoriasis, and 10 samples for severe and 37 for mild atopic dermatitis. The mean abundance of each cellular subset is plotted for mild and severe cases of psoriasis (Fig. 3d) and atopic dermatitis (Fig. 3e). Severe cases of psoriasis showed a significant increase (corrected *p* < 0.05) in the abundance of adipose stem cells and keratinocytes compared to mild cases. Further, a significant decrease (corrected *p* < 0.05) in the relative fraction of adipocytes and endothelial cells was observed for severe psoriasis. In severe cases of atopic dermatitis, only adipose stem cells showed a significant decrease (corrected *p* < 0.05) in their relative fraction.

**Fig. 4:**
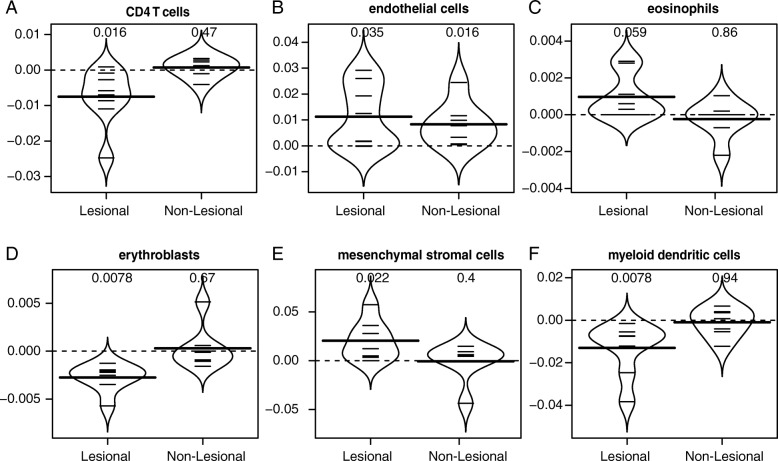
Changes in cellular composition due to narrow-band UVB phototherapy in atopic dermatitis. Comparison of the abundance of CD4^+^ T cells (**a**), endothelial cells (**b**), eosinophils (**c**), erythroblasts (**d**), mesenchymal stromal cells (**e**), and myeloid dendritic cells (**f**) in the lesional and non-lesional skin of atopic dermatitis patients before and after narrow-band UVB phototherapy. The y-axis of each panel corresponds to the difference in cell fraction before vs. after treatment). The *p*-values of each comparison are presented above each beanplot. Expression data from dataset GSE27887 [37] was used for this analysis. Results for other cell types can be found in Additional file 7: Figure S4

**Fig. 5:**
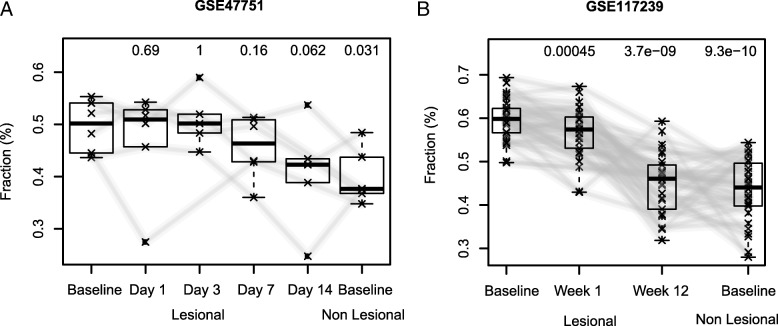
Reduction in the abundance of keratinocytes upon treatment of psoriasis with Etanercept. Comparison of the abundance of keratinocytes in lesional and non-lesional skin before, during, and after treatment with a biologic agent using expression data from two different datasets, i.e. GSE47751 [35] (**a**) and GSE117239 [36] (**b**). The *p*-values of each comparison are presented above each box in the boxplots. Results for other cell types can be found in Additional files 8: Figure S5 and Additional file 9: Figure S6

An important application of deconvolution methodologies in the field of dermatology is the tracing of treatment progress. Here, we used our signature matrix to deconvolve expression data from samples of atopic dermatitis (GSE27887), shined with narrow-band ultraviolet light B (UVB) treatment (Fig. 4), and psoriasis (GSE47751 and GSE117239), treated with Etanercept (Fig. 5). The dataset on atopic dermatitis (GSE27887) comprises expression data on lesional and non-lesional skin biopsy samples before and after phototherapy (Fig. 4 and Additional file 7: Figure S4) [37]. The datasets on psoriasis contain expression data on biopsy samples from lesional skin before, during, and after treatment with a biologic agent targeting TNF-α, as well as samples from non-lesional skin at baseline (Fig. 5 and Additional file 8: Figure S5 and Additional file 9: Figure S6) [35, 36]. Fig. 4 depicts the fold change in the relative fraction of CD4^+^ T cells, endothelial cells, eosinophils, erythroblasts, mesenchymal stromal cells, and myeloid dendritic cells in the lesional and non-lesional skin before and after treatment. Our results suggested that narrow-band UVB phototherapy induces stronger changes in the cellular composition of lesional skin than in non-lesional skin. In the lesional skin, the relative fraction of myeloid dendritic cells, erythroblasts, and CD4^+^ T cells were reduced due to the treatment, while the abundance of endothelial cells and mesenchymal stromal cells increased (without correction for multiple testing). Non-lesional skin irradiated with narrow-band UVB did not result in any significant changes except for an increase in endothelial cells as compared to before phototherapy. Figure 5 shows the estimated fraction of keratinocytes for the samples on datasets GSE47751 and GSE117239 at baseline and specific timepoints of treatment with Etanercept, a biologic agent that hinders the synergy between TNF-α and IL-17A and results in reduced inflammation levels and a lower abundance of keratinocytes [35, 36]. Our results showed a clear reduction of the keratinocytes fraction when treated with Etanercept. The estimated keratinocytes fraction at the end of the treatment was similar to the predicted abundance of this cell type in non-lesional skin. Cell types associated with inflammation also returned to values similar to those estimated for non-lesional skin (Additional file 8: Figure S5 and Additional file 9: Figure S6).

## Discussion

To simplify the interpretation of skin transcriptomics data and enable the study of cellular composition, previous investigations have identified mRNA expression signatures of skin-specific cell types, structures, and processes [14, 15]. However useful, these resources do not allow for the quantitative analysis of the numerical variations of multiple cell types in a skin sample. In this study, we designed DerM22, a signature matrix that includes the mRNA expression signatures of skin-specific and immune cell types. We used this signature matrix to quantify changes in the cellular abundance of the skin in health and chronic inflammatory dermatological disorders. We show that the characteristic inflammatory hallmark and structural changes associated with the lesional skin of these diseases are reflected in the relative fractions derived from the deconvolution of whole skin- expression data from 648 samples. We observed a marked increase in the relative abundance of keratinocytes and leukocytes in the lesions of psoriasis and a small non-significant increase in AD lesions. Further, our results suggest the presence of adipose tissue cells within the skin layers. Interestingly, the relative fraction of these cells varies from healthy to diseased skin, and from non-lesional to lesional skin.

Human skin comprises a complex mix of cell types, the abundance of which is altered in psoriasis and atopic dermatitis [51, 52]. Psoriasis is characterized by the hyperproliferation and disturbed differentiation of keratinocytes, which results in the thickening of the epidermis [53]. This trait is due to the increased levels of IL-17, which induce a self-amplifying inflammatory response in the keratinocytes that drives the development of the psoriasis lesions and the recruitment of leukocytes into the skin [54]. These immune infiltrates include dendritic cells, macrophages, and T cells [55, 56]. In agreement with this, we observed a significant increase in the relative abundance of keratinocytes, T cells, monocytes, plasmacytoid dendritic cells, and to a lesser extent macrophages in the lesional skin of psoriasis compared to the non-lesional skin (Fig. 3a-c). Lesions of atopic dermatitis contain a large number of resident and infiltrated immune cells, e.g., dendritic cells, eosinophils, and T cells [57]. Our results indicated that the relative fraction of these cell types increases considerably in the lesional skin of atopic dermatitis, compared to the non-lesional and healthy skin (Fig. 3b). Suárez Fariñas et al. [10] previously reported that non-lesional skin is distinct from healthy skin concerning epidermal differentiation and immune abnormalities. They described that non-lesional AD skin has a variable immune phenotype, which depends on the severity and extent of the disease. In contrast with their observations, our results suggested a tendency towards a lower fraction of immune cells in the uninvolved skin of atopic dermatitis compared to the healthy skin (Fig. 3b), although we could not establish statistical significance when comparing healthy vs. uninvolved atopic dermatitis samples when comparing samples within studies (Fig. 3c). Further studies need to establish whether this lacking significance is due to the limited power in our analyses or whether the effect seen in Fig. 3a,b is due to incorporation of different datasets for healthy individuals and individuals with atopic dermatitis, which may result in a slight bias due to batch effects, although the influence of batch effects is only small (Fig. 1d, Additional file 5: Figure S2).

One intriguing finding in our results is the abundance of adipose stem cells and adipocytes predicted for the skin in health and disease. Figure 2 shows a low but present fraction of adipose stem cells in the epidermal layer and sublayers. Chavez-Munoz et al. [58] described the trans-differentiation of adipose stem cells into keratinocytes, which could potentially explain the observed fraction of adipose stem cells in the epidermal layer. The deconvolution of data from isolated cell types (Fig. 1c) indicated that there is a minor overlap in the expression of keratinocytes and adipose stem cells. The role of adipose stem cells in the skin has not been fully elucidated yet. However, it has been suggested that adipose stem cells may contribute to the maintenance of a healthy epidermis and dermis [59]. The inferred tissue composition derived from the expression data of whole-skin biopsies (Fig. 3a) also suggested the presence of adipose stem cells in the skin. It has been shown that mesenchymal stem cells are involved in the immunomodulation of lymphocytes in the skin [60]. They can inhibit the proliferation and differentiation of these immune cells through the induction of cell cycle arrest, cell-to-cell contact, secretion of soluble mediators, and the regulation of dendritic cells and monocytes [60]. Psoriasis and atopic dermatitis involve impaired immunoregulation [61], which could be related to the lower amount of adipose stem cells in both diseases compared to the healthy skin. Adipocytes were predicted to exist in the epidermal and dermal layers of the skin, with a higher relative fraction in the dermis compared to the epidermis. The presence of adipocytes in the dermis has been described in the literature [62, 63], but it does not entirely explain the predicted abundance. Adipocytes were also observed in the results from the whole-skin biopsies deconvolution (Fig. 3a), particularly in the non-lesional skin of atopic dermatitis. In the lesional skin of psoriasis, the estimated abundance of adipocytes was the lowest in comparison to non-lesional psoriasis, atopic dermatitis, and healthy skin. We propose two factors that may contribute to these trends. (i) In skin diseases, the increased contribution of keratinocytes and immune cells would decrease the fractions of adipose stem cells and adipocytes accordingly. (ii) The punch biopsies from which the expression data was derived may have had a varying depth depending on the location of sample acquisition, and the state and severity of the disease. This would result in samples with varying content of adipose tissue. Further studies are needed to discriminate between these possibilities and investigate the role of adipose cell types in chronic inflammatory dermatological disorders.

Another unexpected finding was the low fraction of CD8^+^ T cells in the epidermis (Fig. 2), i.e. below 1%. Previous studies have reported an abundance of CD8^+^ T cells in the epidermis [64]. These T cells have been observed at the borderline of the epidermis and the dermis in humans in healthy or psoriatic skin [65]. The discrepancy of our results with those reported in the literature may be due to the method of sample acquisition, i.e. laser capture microdissection, which was used to isolate the layer-specific skin samples. In agreement with literature, we observed a higher fraction of CD4^+^ T cells in the dermis [64]. Despite being predominantly found in the dermis, CD4^+^ T cells have also been observed in the epidermis [65]. Future studies should aim to quantify the abundance of these cellular subsets within the skin layers in health and disease.

Our deconvolution analysis yields consistent results across independent skin-expression datasets (Fig. 1c) and shows a good performance when compared to immunohistochemistry cell counts (Fig. 1b) and flow cytometry data from immune infiltrates (Fig. 1a). Despite the positive results regarding the validation of the methodology, it remains limited. Future efforts should be dedicated to generating experimental data that could aid in the additional validation of DerM22. Further, our proposed methodology has two fundamental limitations. (i) The first limitation is the fidelity of the reference profiles. We tried to address this issue by using a wide range of samples for each cell type. However, it remains a significant constraint, mainly due to the known expression changes in the disease state [14]. Recently, Shih et al. [15] defined 20 gene signatures identified solely from skin-derived expression data. Their set of gene signatures included smooth muscle, adipocytes, fibroblasts, keratinocytes, T cells, and macrophages/DC. These cell types are also included in our signature matrix, but in contrast with them, we included other leukocyte subsets and were able to quantify the abundance of each cell type included in DerM22. (ii) The second limitation is the accurate deconvolution of cross-platform data [23]. We assessed this issue by comparing the results achieved for expression data on the same platform as DerM22 with data of a different platform (Fig. 1a-b). It was evident that the deconvolution of cross-platform data led to similar relative fractions (Fig. 1a) and that isolated cell types were adequately identified (Fig. 1b). However, one should still be cautious when dealing with cross-platform data.

## Conclusions

Here, we have defined a signature matrix that enables the direct enumeration of the different cell-types in whole-skin and skin layer-specific composition based on mRNA expression data. By analyzing mRNA expression data of 648 subjects, we observed and quantified the characteristic pathological changes of the increased number of keratinocytes and infiltration of immune cells associated with psoriasis and atopic dermatitis. Overall, we show that deconvolution-based methodologies can be used to interpret transcriptomic data from whole skin samples and provide insight into the pathological changes associated with skin diseases and their treatment.

## Additional files

Additional file 1: **Table S2.** Signature matrix DerM22. (XLSX 654 kb)

Additional file 2: **Figure S1.** Correlation heatmap of the cellular subsets included in DerM22. (PDF 1013 kb)

Additional file 3: **Table S3.** Reference dataset for DerM22. (XLSX 17 kb)

Additional file 4: **Table S1.** Table of flow cytometry data used in this study. (DOCX 21 kb)

Additional file 5: **Figure S2.** Variation in relative abundance of skin related cell types across independent datasets. The relative fraction of 13 different cell types in the skin was derived from nine independent datasets. These datasets comprised samples of healthy skin and skin from patients with atopic dermatitis (AD) and psoriasis (PS). The disease samples accounted for lesional (L) and non-lesional skin (NL). The standard deviation is derived for each data point in the panels of this figure. (PDF 461 kb)

Additional file 6: **Figure S3.** Comparison of the changes in cellular abundance between lesional and non-lesional skin of patients with psoriasis and atopic dermatitis. The *p*-value (without multiple testing correction) of each comparison is depicted on the top of each bean plot. (PDF 4401 kb)

Additional file 7: **Figure S4.** Changes in cellular composition due to UVB phototherapy. Comparison of the abundance of various cell types in the lesional and non-lesional skin of patients with atopic dermatitis before and after narrow-band UVB phototherapy. Expression data from dataset GSE27887 [35] was used for this analysis. The p-value of each comparison is presented above each beanplot. (PDF 863 kb)

Additional file 8: **Figure S5.** Changes in cellular composition due to Etanercept treatment before, during, and after treatment. Comparison of the abundance of various cell types in the lesional and non-lesional skin of patients with psoriasis. Expression data from dataset GSE47751 was used for this analysis. The *p*-values of each comparison are presented above each box in the boxplots. (PDF 701 kb)

Additional file 9: **Figure S6.** Changes in cellular composition due to Etanercept treatment at baseline and treatment weeks 1 and 12. Comparison of the abundance of various cell types in the lesional and non-lesional skin of patients with psoriasis. Expression data from dataset GSE17239 was used for this analysis. The p-values of each comparison are presented above each box in the boxplots. (PDF 2240 kb)

## Data Availability

The details on the data used for the development of the signature matrix DerM22 utilized in the current study is available in the Additional file 3: Table S3. The signature matrix is available in the Additional file 1: Table S2. The datasets analyzed in the present study are available in the ArrayExpress repository with accession number E-MEXP-750, and the Gene Expression Omnibus database with accession numbers GSE42114, GSE13355, GSE30999, GSE34248, GSE41662, GSE78097, GSE14905, GSE47751, GSE117239, GSE27887, GSE32924, GSE36842, GSE6710, GSE22886, GSE4527, GSE5099, GSE7138, GSE26688, GSE6932, GSE4858.

## Abbreviations

AD: Atopic dermatitis
FC: Flow cytometry
mDCs: Myeloid dendritic cells
NK: Natural killer cells
pDCs: Plasmacytoid dendritic cells
pp: Percentage points
Ps: Psoriasis
SCs: Stem cells
TAs: Transit amplifying cells
UVB: Ultraviolet light B
