# Supplementary figures and images for: Characterization of disease-specific cellular abundance profiles of chronic inflammatory skin conditions from deconvolution of biopsy samples
Source: BMC Med Genomics. 2019 Aug 17;12:121. doi: 10.1186/s12920-019-0567-7 (PMC6698047; doi:10.1186/s12920-019-0567-7)

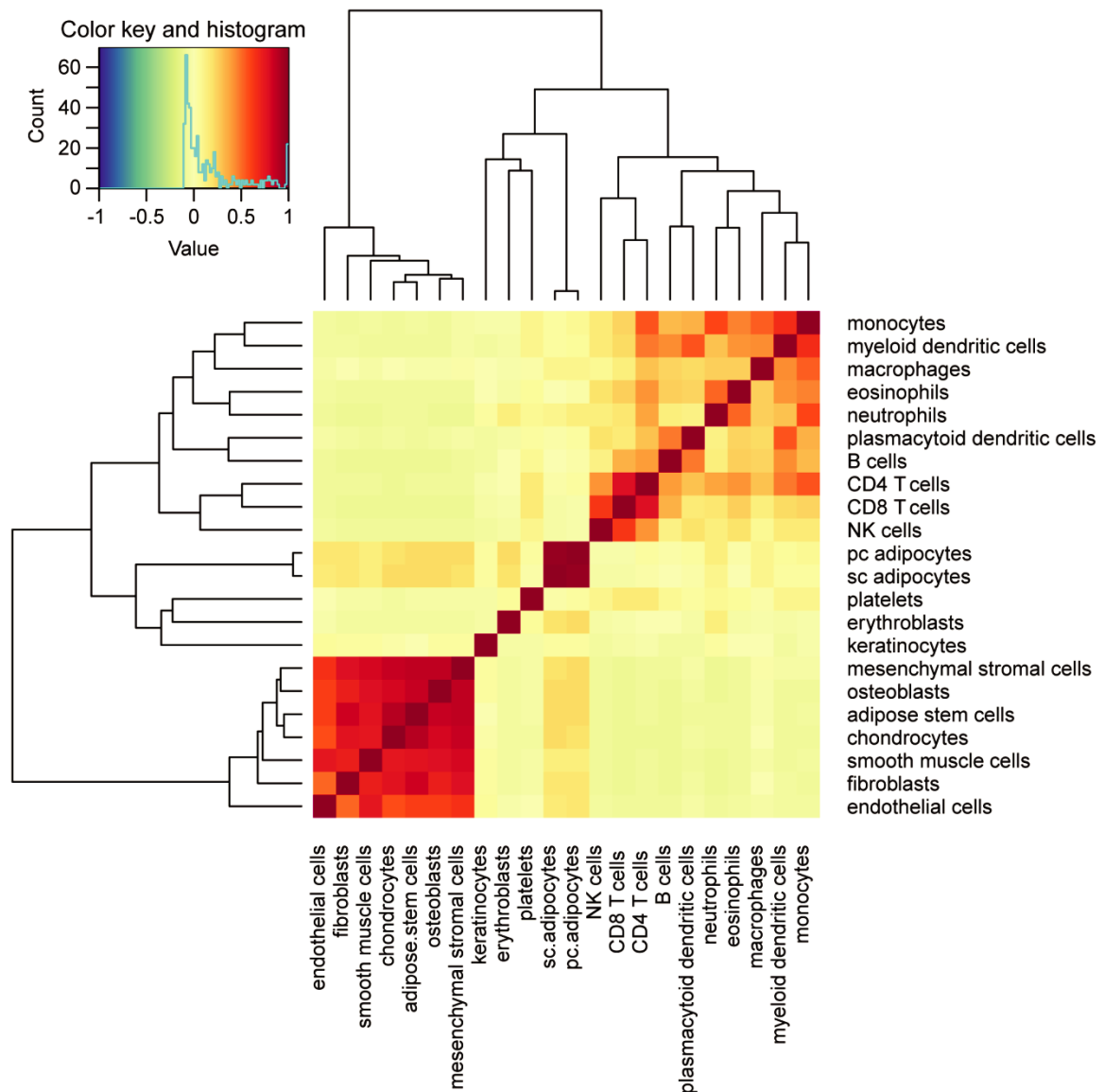

**Fig. S1. Correlation of cellular subsets included in Derm22.**

Supplement: Supplementary file 2 — Figure S1. Correlation heatmap of the cellular subsets included in DerM22. (PDF 1013 kb) [file 12920_2019_567_MOESM2_ESM.pdf]

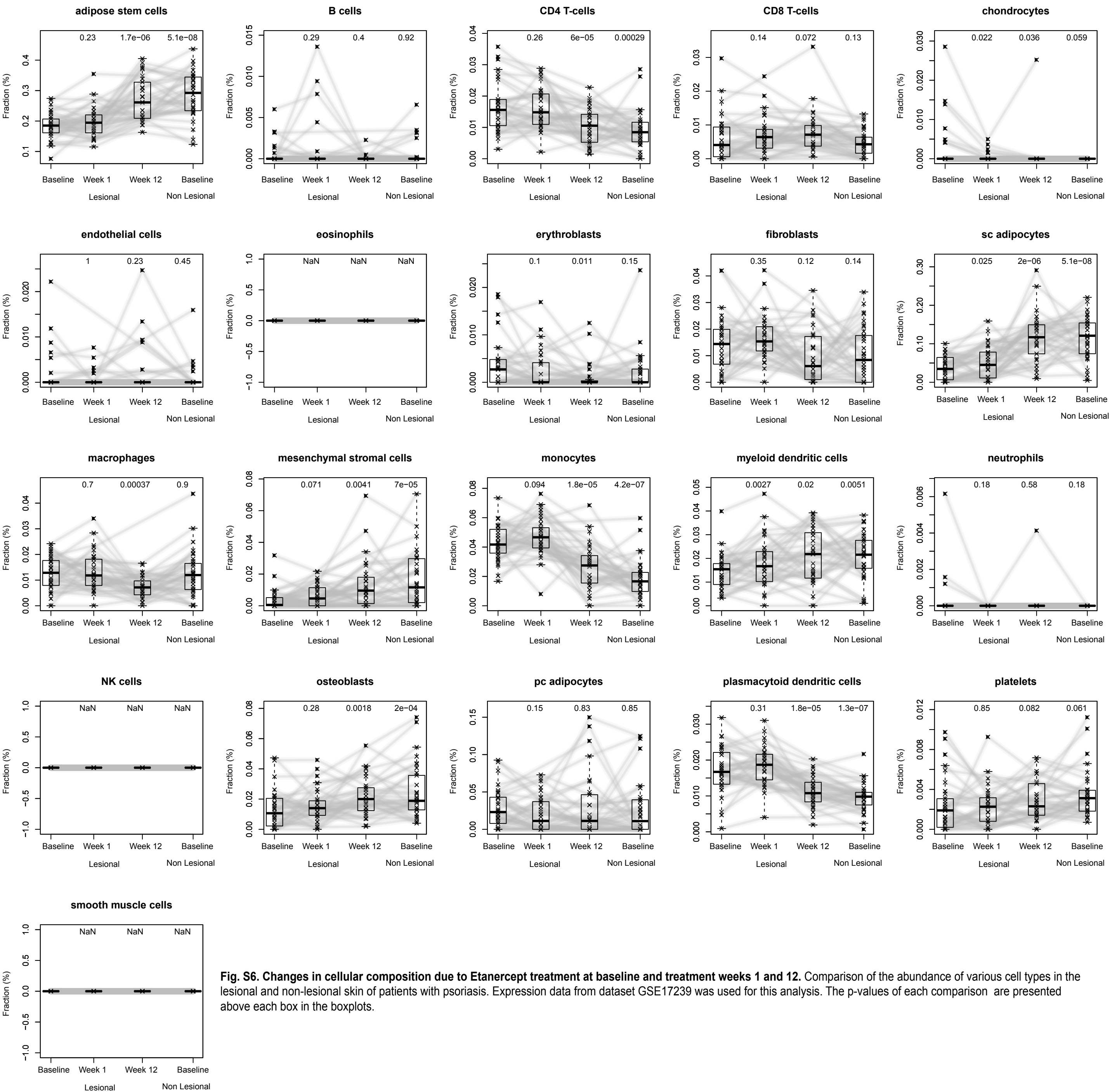

Supplement: Supplementary file 9 — Figure S6. Changes in cellular composition due to Etanercept treatment at baseline and treatment weeks 1 and 12. Comparison of the abundance of various cell types in the lesional and non-lesional skin of patients with psoriasis. Expression data from dataset GSE17239 was used for this analysis. The p-values of each comparison are presented above each box in the boxplots. (PDF 2240 kb) [file 12920_2019_567_MOESM9_ESM.pdf]
