# Supplementary material for: Characterization of disease-specific cellular abundance profiles of chronic inflammatory skin conditions from deconvolution of biopsy samples
Source: BMC Med Genomics. 2019 Aug 17;12:121. doi: 10.1186/s12920-019-0567-7 (PMC6698047; doi:10.1186/s12920-019-0567-7)
Supplement: Supplementary file 4 — Table S1. Table of flow cytometry data used in this study. (DOCX 21 kb) [file 12920_2019_567_MOESM4_ESM.docx]

Table S1. Flow cytometry data considered in this study.

|  | **Healthy** | | **Psoriasis Lesional** | |
| --- | --- | --- | --- | --- |
| **Class** | **Flow cytometry** | **Ref** | **Flow cytometry** | **Ref** |
| B cells (CD19+) |  | | 4.3+-.2 | [1] |
| pDCs (BDCA-2+ CD123++) | 0.03 | [2] | 8.6 | [2] |
| keratinocytes (Ker 10+) | 57.8+-3.4 | [3] | 44.3+-4.4 | [4] |
|  | | | 41.0+-5.3 | [4] |
|  |  |  | 35.7+-3.3 | [4] |
|  |  |  | 37.9+-4.2 | [4] |
|  |  |  | 35.2+-3 | [5] |
|  |  |  | 37.7+-3.7 | [5] |
| infiltrates (Vimentin+) | 7.2+-.6 | [3] | 10.9+-2.2 | [4] |
|  | | | 10.8+-2 | [4] |
|  |  |  | 14.4+-1.8 | [4] |
|  |  |  | 15.5+-2.8 | [4] |
|  |  |  | 15.1+-2.4 | [5] |
|  |  |  | 16.2+-2.5 | [5] |

1. Lu J, Ding Y, Yi X, Zheng J, Lu J, Ding Y, et al. CD19+ B cell subsets in the peripheral blood and skin lesions of psoriasis patients and their correlations with disease severity. Braz J Med Biol Res. 2016;49. doi:10.1590/1414-431X20165374.

2. Nestle FO, Conrad C, Tun-Kyi A, Homey B, Gombert M, Boyman O, et al. Plasmacytoid predendritic cells initiate psoriasis through interferon-α production. J Exp Med. 2005;202:135–43. doi:10.1084/jem.20050500.

3. Glade CP, van Erp PE, van Hooijdonk CA, Elbers ME, van de Kerkhof PC. Topical treatment of psoriatic plaques with 1 alpha, 24 dihydroxyvitamin D3: a multiparameter flow cytometrical analysis of epidermal growth, differentiation and inflammation. Acta Derm Venereol. 1995;75:381–5.

4. Glade C p., Van Erp P e. j., Van De Kerkhof P c. m. Epidermal cell DNA content and intermediate filaments keratin 10 and vimentin after treatment of psoriasis with calcipotriol cream once daily, twice daily and in combination with clobetasone 17-butyrate cream or betamethasone 17-valerate cream: a comparative flow cytometric study. Br J Dermatol. 1996;135:379–84. doi:10.1046/j.1365-2133.1996.d01-1008.x.

5. Raxworthy MJ, Cunliffe WJ, Wood EJ. The influence of proteases on the colony-forming efficiency of human keratinocytes in culture. Biochem Soc Trans. 1987;15:519–20. doi:10.1042/bst0150519.
