# Supplementary material for: Characterization of disease-specific cellular abundance profiles of chronic inflammatory skin conditions from deconvolution of biopsy samples
Source: BMC Med Genomics. 2019 Aug 17;12:121. doi: 10.1186/s12920-019-0567-7 (PMC6698047; doi:10.1186/s12920-019-0567-7)
Supplement: Supplementary file 5 — Figure S2. Variation in relative abundance of skin related cell types across independent datasets. The relative fraction of 13 different cell types in the skin was derived from nine independent datasets. These datasets comprised samples of healthy skin and skin from patients with atopic dermatitis (AD) and psoriasis (PS). The disease samples accounted for lesional (L) and non-lesional skin (NL). The standard deviation is derived for each data point in the panels of this figure. (PDF 461 kb) [file 12920_2019_567_MOESM5_ESM.pdf]

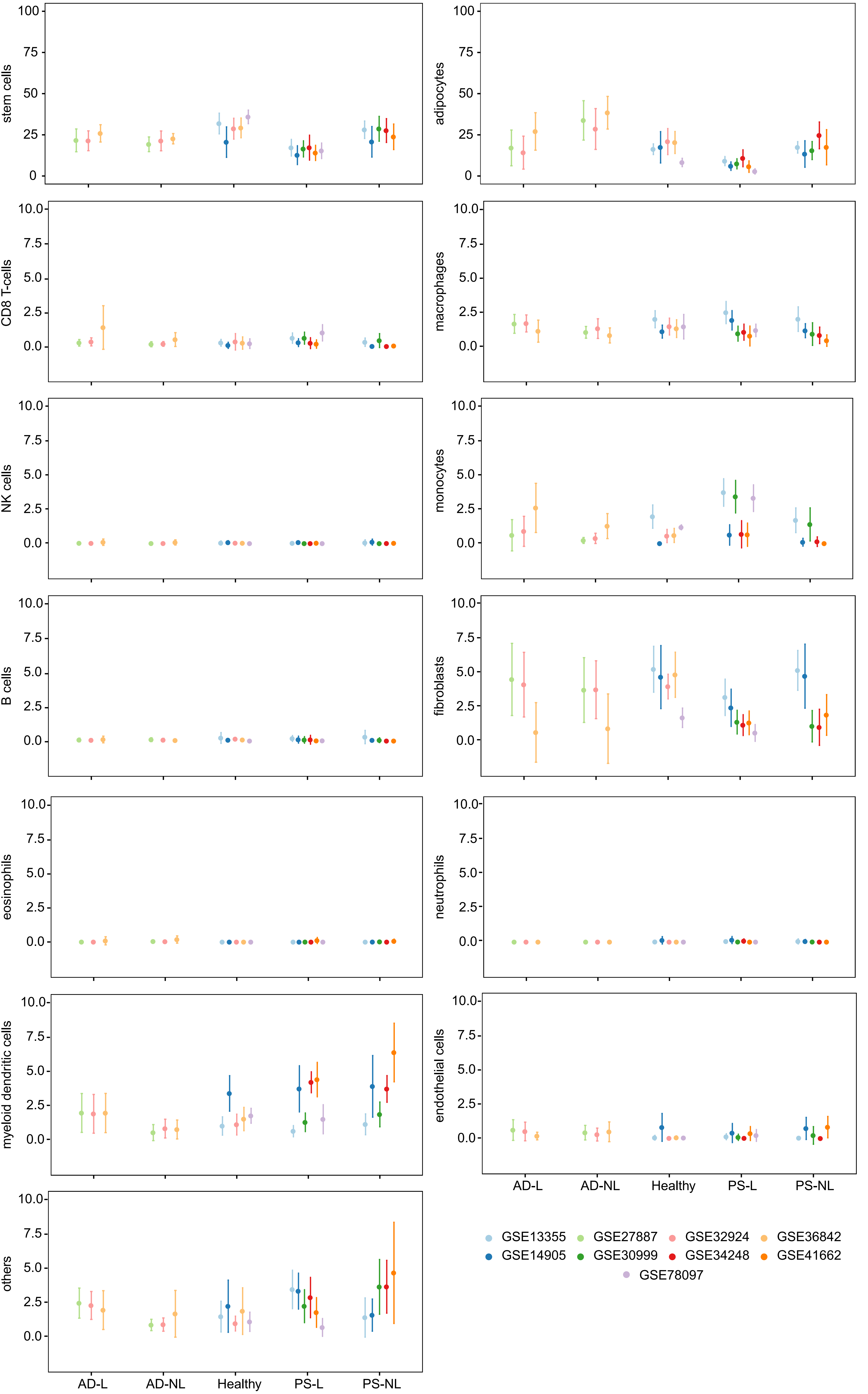

Fig. S2. Variation in relative abundance of skin related cell types across independent datasets. The relative fraction of 13 different cell types in the skin was derived from nine independent datasets. These datasets comprised samples of healthy skin and skin from patients with atopic dermatitis (AD) and psoriasis (PS). The disease samples accounted for lesional (L) and non-lesional skin (NL). The standard deviation is derived for each data point in the panels of this figure.
