# Supplementary material for: Characterization of disease-specific cellular abundance profiles of chronic inflammatory skin conditions from deconvolution of biopsy samples
Source: BMC Med Genomics. 2019 Aug 17;12:121. doi: 10.1186/s12920-019-0567-7 (PMC6698047; doi:10.1186/s12920-019-0567-7)
Supplement: Supplementary file 6 — Figure S3. Comparison of the changes in cellular abundance between lesional and non-lesional skin of patients with psoriasis and atopic dermatitis. The p-value (without multiple testing correction) of each comparison is depicted on the top of each bean plot. (PDF 4401 kb) [file 12920_2019_567_MOESM6_ESM.pdf]

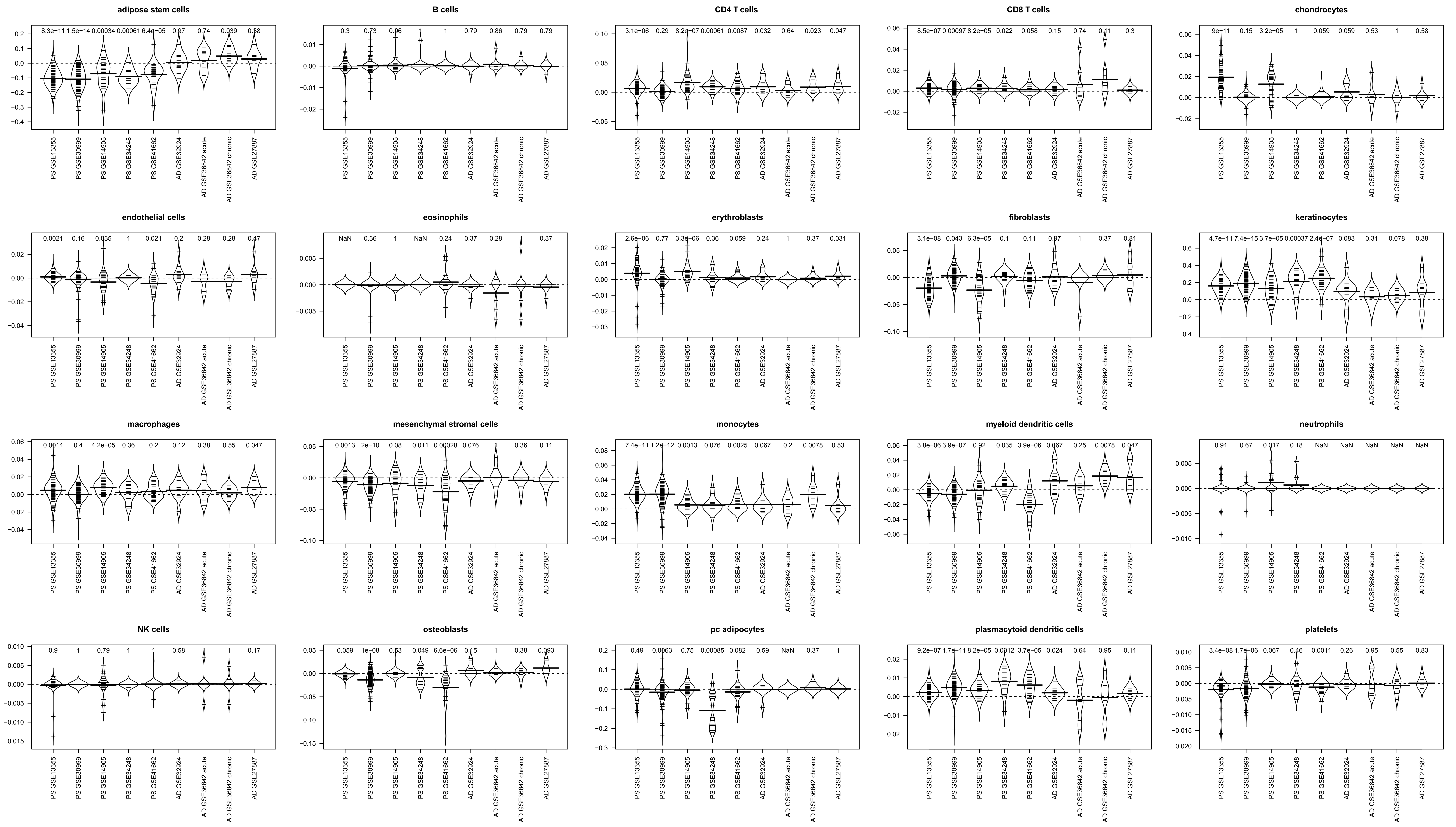

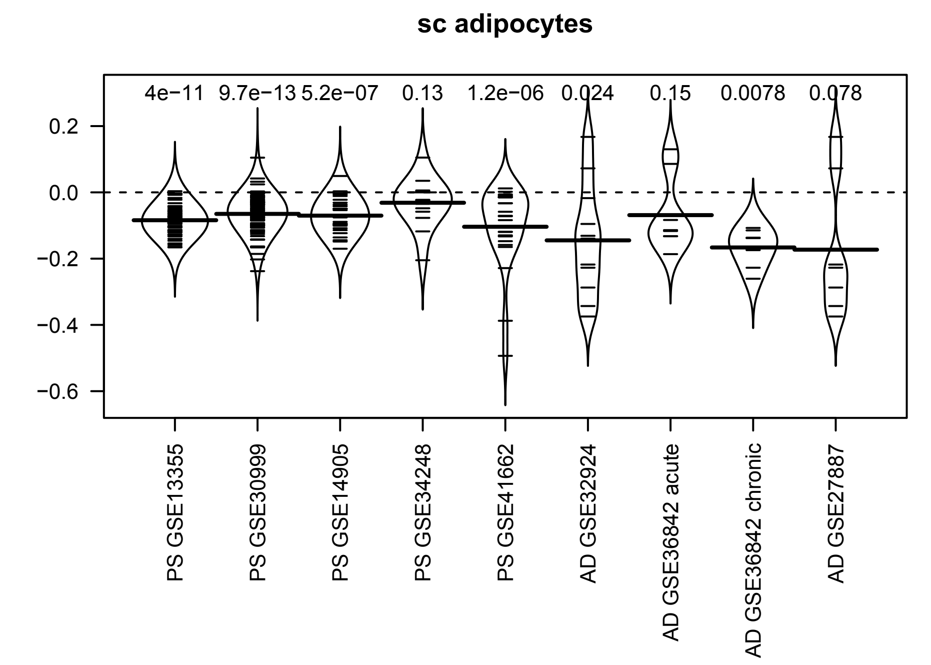

Fig. S3. Comparison of the changes in cellular abundance between lesional and non-lesional skin of patients with psoriasis (PS) and atopic dermatitis (AD). The p-value of each comparison is depicted on the top of each bean plot.
