# Supplementary material for: Characterization of disease-specific cellular abundance profiles of chronic inflammatory skin conditions from deconvolution of biopsy samples
Source: BMC Med Genomics. 2019 Aug 17;12:121. doi: 10.1186/s12920-019-0567-7 (PMC6698047; doi:10.1186/s12920-019-0567-7)
Supplement: Supplementary file 7 — Figure S4. Changes in cellular composition due to UVB phototherapy. Comparison of the abundance of various cell types in the lesional and non-lesional skin of patients with atopic dermatitis before and after narrow-band UVB phototherapy. Expression data from dataset GSE27887 [35] was used for this analysis. The p-value of each comparison is presented above each beanplot. (PDF 863 kb) [file 12920_2019_567_MOESM7_ESM.pdf]

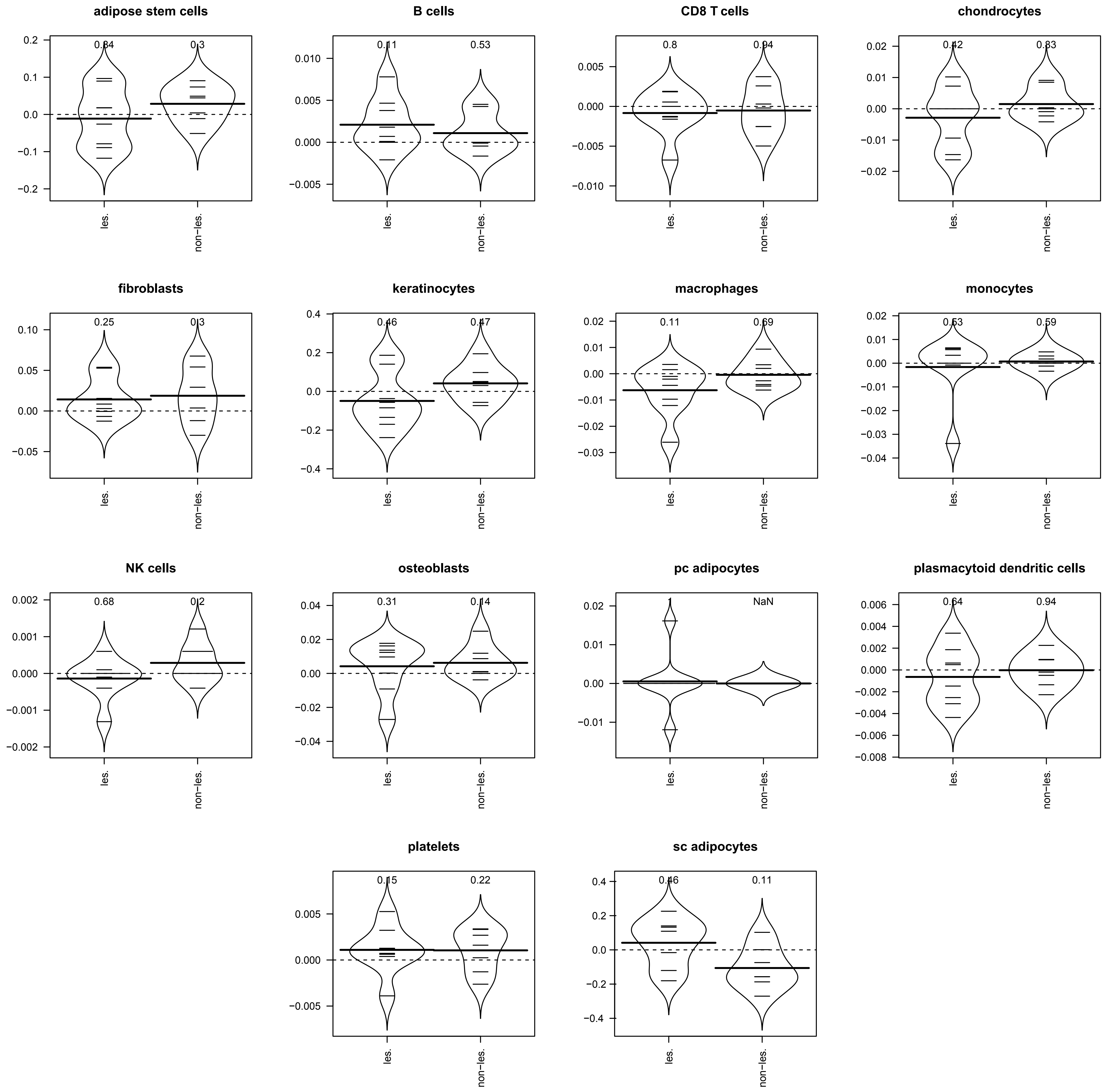

Fig. S4. Changes in cellular composition due to UVB phototherapy. Comparison of the abundance of various cell types in the lesional (les) and non-lesional (non-les) skin of patients with atopic dermatitis before and after narrow-band UVB phototherapy. Expression data from dataset GSE27887 was used for this analysis. The p-values of each comparison are presented above each beanplot.
