# Supplementary material for: Characterization of disease-specific cellular abundance profiles of chronic inflammatory skin conditions from deconvolution of biopsy samples
Source: BMC Med Genomics. 2019 Aug 17;12:121. doi: 10.1186/s12920-019-0567-7 (PMC6698047; doi:10.1186/s12920-019-0567-7)
Supplement: Supplementary file 8 — Figure S5. Changes in cellular composition due to Etanercept treatment before, during, and after treatment. Comparison of the abundance of various cell types in the lesional and non-lesional skin of patients with psoriasis. Expression data from dataset GSE47751 was used for this analysis. The p-values of each comparison are presented above each box in the boxplots. (PDF 701 kb) [file 12920_2019_567_MOESM8_ESM.pdf]

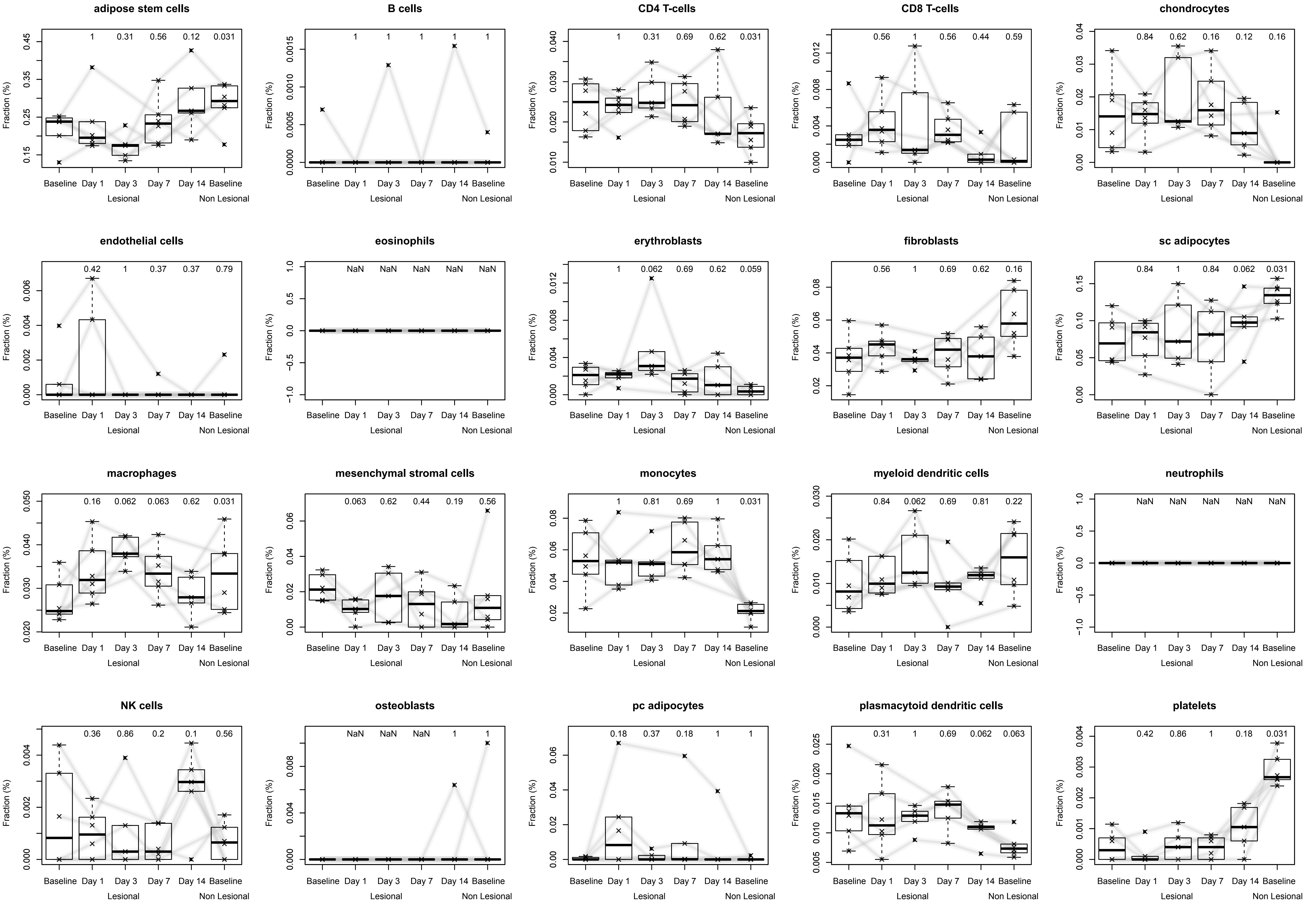

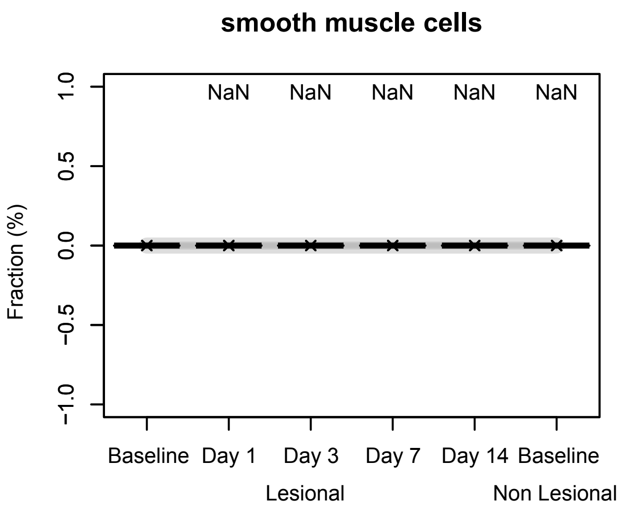

Fig. S5. Changes in cellular composition due to Etanercept treatment. Comparison of the abundance of various cell types in the lesional and non-lesional skin of patients with psoriasis before, during, and after treatment. Expression data from dataset GSE47751 was used for this analysis. The p-values of each comparison are presented above each box in the boxplots.
